# Clonal evolution driven by superdriver mutations

**DOI:** 10.1186/s12862-020-01647-y

**Published:** 2020-07-20

**Authors:** Patrick Grossmann, Simona Cristea, Niko Beerenwinkel

**Affiliations:** 1 Department of Biosystems Science and Engineering, ETH Zurich, Mattenstrasse 26, 4058 Basel, Switzerland; 2 Department of Biostatistics & Computational Biology, Dana-Farber Cancer Institute, Boston, MA USA; 3 Department of Biostatistics, Harvard T.H. Chan School of Public Health, Boston, MA USA; 4 Harvard Department of Stem Cell and Regenerative Biology, Cambridge, MA USA; 5 SIB Swiss Institute of Bioinformatics, 4058 Basel, Switzerland

**Keywords:** Cancer progression, Tumorigenesis, Mutation, Selection, Fitness, Waiting time to cancer

## Abstract

**Background:**

Tumors are widely recognized to progress through clonal evolution by sequentially acquiring selectively advantageous genetic alterations that significantly contribute to tumorigenesis and thus are termned drivers. Some cancer drivers, such as *TP53* point mutation or *EGFR* copy number gain, provide exceptional fitness gains, which, in time, can be sufficient to trigger the onset of cancer with little or no contribution from additional genetic alterations. These key alterations are called superdrivers.

**Results:**

In this study, we employ a Wright-Fisher model to study the interplay between drivers and superdrivers in tumor progression. We demonstrate that the resulting evolutionary dynamics follow global clonal expansions of superdrivers with periodic clonal expansions of drivers. We find that the waiting time to the accumulation of a set of superdrivers and drivers in the tumor cell population can be approximated by the sum of the individual waiting times.

**Conclusions:**

Our results suggest that superdriver dynamics dominate over driver dynamics in tumorigenesis. Furthermore, our model allows studying the interplay between superdriver and driver mutations both empirically and theoretically.

## Background

Tumorigenesis is widely recognized as an evolutionary process resulting from the sequential accumulation of genetic alterations. Many of these alterations occur in onco- and tumor suppressor genes, as well as in genes regulating the DNA repair or replication mechanisms [1–3].

Mathematical modeling of tumorigenesis has a rich history and seeks to describe the evolutionary dynamics of tumor growth and mutation accumulation [4, 5]. The initial two-hit and multi-stage theories [6–9] suggested early on that multiple mutations leading to cancer are acquired sequentially over large periods of time. This hypothesis then evolved into more elaborate models in discrete and continuous time [10–13], supported by a substantial body of empirical evidence [2, 14–16].

A large fraction of these models follow the theory of clonal evolution, according to which some genetic alterations (commonly referred to here as *mutations*) confer the hosting cell with significant increases in selective fitness [4]. These mutations are called driver mutations and the genes they affect are called driver genes. The fitness increase enables the cell to produce relatively more offspring than cells without the driver mutation through various biological mechanisms such as resistance to apoptosis or accelerated proliferation. Other types of mutations have been considered in the modeling literature, such as passenger and deleterious mutations, which are selectively neutral and confer a fitness disadvantage, respectively [17, 18].

Various stochastic models of clonal evolution have been suggested to study tumorigenesis, especially using the related Moran and Wright-Fisher models [19–22]. In particular, Beerenwinkel et al. and Bozic et al. [14, 21] proposed Wright-Fisher [23, 24] models with driver mutations, with the goal of estimating the waiting time to cancer and understanding the role of drivers in tumorigenesis. While the acquisition and accumulation of driver mutations is recognized to lead to the onset and progression of cancer, some critical driver genes, such as *EGFR*, *TP53*, or *KRAS*, are known to dramatically accelerate cancer progression by, for instance, elevating cell proliferation or avoiding apoptosis [2, 25, 26]. There have been early indications that a few rare but highly advantageous mutations may particularly drive the progression of cancer [27]. Hence, explicit modeling of these highly selective mutations, in addition to normal driver mutations, would provide more accurate insights into the study of tumorigenesis as a clonal evolution process.

Here, we introduce the concept of *superdrivers*, an aggressive type of driver mutations that is highly selectively advantageous for a mutated cell through a strongly elevated fitness gain. Examples of superdrivers include *TP53* point mutations or *EGFR* copy number gains across multiple cancer types. We present a discrete-time Wright-Fisher stochastic model to study the evolutionary dynamics of superdrivers in combination with common drivers by extensively simulating tumorigenesis under a wide range of parameters. Moreover, we propose an analytical approximation for the expected waiting time to the first mutated cell with defined numbers of superdrivers and drivers. Our model aims at understanding the evolutionary dynamics of the interplay between superdrivers and drivers in the progression to cancer.

## Methods

### Tumor evolution model

We model tumorigenesis as a Wright-Fisher process with mutation and selection, including two types of selectively advantageous mutations: drivers and superdrivers. Drivers have selective advantage *s* ∊ [0,1], while superdrivers have a *c* times higher selective advantage *r* = *c s,* with superdriver fitness increase parameter *c* > 1. Every driver and superdriver confers the same fitness increase of 1 + *s* and 1 + *r*, respectively, to the cell. This assumption of constant fitness increase captures fitness differences between selectively advantages mutations and it facilitates revealing the fundamental principles of the interplay between both fitness classes. With the addition of superdrivers, our model can be regarded as an extension of the model in [21]. We model tumor growth over *T* = 4500 discrete cell generations, which roughly equals 12 years, assuming one cell division per day. In every generation *t*, the population size *N*(*t*) of the tumor is multiplied by *α* = exp. [log(*N*(*T*)/*N* (0)) / *T*] to obtain *N*(*t* + 1), where we consider initial and final population sizes of *N* (0) = 10^6^ and *N*(*T*) = 10^9^ cells, respectively. In our simulations, each cell can acquire at most *n* = 10 superdriver and *m* = 100 driver mutations. Initially, all cells are modeled without any mutated loci. Assuming that fitness effects of mutations are multiplicative, the relative fitness *ω*_*kl*_ of a cell with *k* superdriver and *ℓ* driver mutations in generation *t* is given by

$$ {\omega}_{k\mathit{\ell}}=\frac{{\left(1+r\right)}^k{\left(1+s\right)}^{\ell }}{\sum_{i=0}^n{\sum}_{j=0}^m{\left(1+r\right)}^i{\left(1+s\right)}^j{x}_{ij}}, $$

where *N*_*ij*_(*t*) is the absolute and *x*_*ij*_ = *x*_*ij*_(*t*) = *N*_*ij*_(*t*)/*N*(*t*) is the relative frequency of the clone with *i* superdriver and *j* driver mutations, where we have suppressed the dependency on *t*. Assuming independent effects of mutations on fitness and no back mutations, the probability of sampling a mutant with *k* superdrivers and *ℓ* drivers, a (*k*, *ℓ*) cell for short, is given by

$$ {\theta}_{k\mathit{\ell}}=\sum \limits_{i=0}^k\sum \limits_{j=0}^{\ell}\left(\genfrac{}{}{0pt}{}{n-i}{k-i}\right)\left(\genfrac{}{}{0pt}{}{m-j}{\ell -j}\right){\mu}^{k-i+\ell -j}{\left(1-\mu \right)}^{n-i+m-j}{\omega}_{k\mathit{\ell}}{x}_{ij} $$

where *μ =* 10^− 8^ is the mutation probability per gene. In every generation, the cell population then is updated according to a multinomial distribution with parameters *θ*^*(t)*^ *=* (*θ*^*(t)*^_*kℓ*_),

$$ \left[{N}_{00}\left(t+1\right),\dots, {N}_{nm}\left(t+1\right)\right]\sim Mult\ \left(\alpha N(t),{\theta}^{(t)}\right), $$

where *Mult* is the multinomial distribution.

### Simulation of tumorigenesis

We simulated tumorigenesis using the model described above by varying the driver selection parameter *s* ∊ {0.005, 0.01, 0.02, 0.03, 0.04, 0.05} and the superdriver factor *c* ∊ {1, 1.1, 1.3, …, 3}, and we report our results based on the mean across 50 replicates. Simulation code was written in C (compiled by GCC version 4.2 on Linux) and statistical analysis was carried out with R (version 3.0.1 on Linux).

### Waiting time analysis

Our simulation results suggest that the expected waiting time τ_*kℓ*_ to a combined set of *k* superdriver and *ℓ* driver mutations can be approximated by the sum of the individual waiting times *T*_*k*_^*S*^ to *k* superdrivers and *T*_*ℓ*_^*D*^ to *ℓ* drivers alone. *T*_*k*_^*S*^ and *T*_*ℓ*_^*D*^ are approximated as in [21] by decoupling selection and mutation, such that

$$ {T_k}^S\approx \frac{k\kern0.5em {\log}^2\left[r/\left(\upmu \kern0.5em n\right)\right]}{r\kern0.5em \log \left[N(t)N(0)\right]}\kern0.5em \mathrm{and}\kern0.5em {T_{\mathrm{\ell}}}^D\approx \frac{\mathrm{\ell}\kern0.5em {\log}^2\left[s/\left(\upmu \kern0.5em m\right)\right]}{s\kern0.5em \log \left[N(t)N(0)\right]}, $$

and hence τ_*kℓ*_ ≈ *T*_*k*_^*S*^*+ T*_*ℓ*_^*D*^*.*

### Error modeling

To understand the agreement between the model simulations and analytical waiting time approximations, we fitted a linear regression model to predict the residual between simulation and approximation, using the following predictors: driver fitness, superdriver fitness factor, number of superdriver mutations to wait for, and number of driver mutations to wait for:

$$ {\boldsymbol{\tau}}_{k\mathrm{\ell}}\approx {T_k}^S+{T_{\mathrm{\ell}}}^D+\varepsilon, $$

where *ε = β*_*0*_ *+ β*_*1*_*s + β*_*2*_*r + β*_*3*_*k + β*_*4*_*ℓ* is the error term with intercept *β*_*0*_ and coefficients *β*_*i*_.

Further, to analyze the relative effects of driver and superdriver fitnesses, we fitted an additional model where *ε = β*_*0*_ *+ β*_*1*_*s + β*_*2*_*c + β*_*3*_*k + β*_*4*_*ℓ,* with *c* = *r*/*s*. We estimated the regression model from simulated data using all combinations of *s* ∊ {0.005, 0.01, 0.02, 0.03, 0.04, 0.05}, *c* ∊ {1, 1.1, 1.3, …, 3}, *k* ∊ {1, …, 6}, and *ℓ* ∊ {1, …, 10}.

## Results

**Fig. 1:**
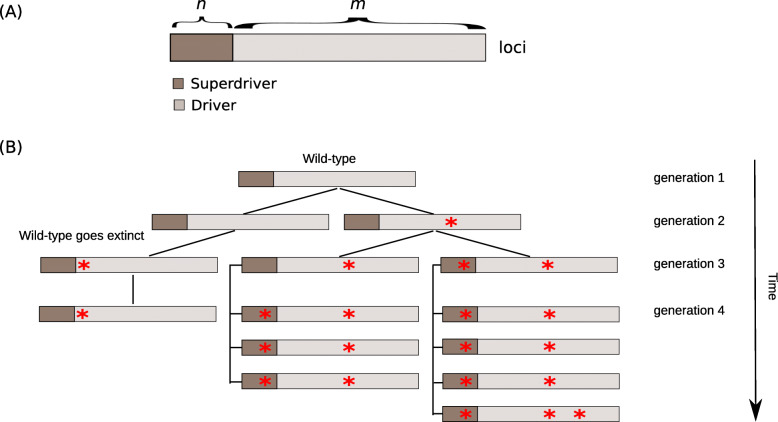
Wright Fisher process of tumor progression. **a** Representation of genotypes. The genotypes of all cells are modeled with *n* = 10 superdriver and *m* = 100 driver loci. Every locus is susceptible to a mutation event (red asterisk) with the same probability in any cell in each generation. **b** Growing population of tumor cells. The system starts with 10^6^ wild-type cells, i.e., genotypes without any mutated loci, and grows exponentially to 10^9^ cells acquiring multiple superdriver and driver mutations. Every mutation increases the fitness of a cell and hence the likelihood of sampling an offspring which will carry that same mutation

To describe the evolutionary dynamics of tumorigenesis with superdrivers and drivers, we employ a Wright-Fisher model. In every generation of tumor cells, a mutation can hit a superdriver or driver locus. Superdrivers are modeled with a selective advantage of *r* = *c s*, where *c >* 1 is the superdriver fitness increase parameter and *s* ∊ [0,1] is the driver advantage. We simulated tumorigenesis of exponentially growing cell populations (Fig. 1); this simulation begins with an unmutated genome in the first generation, which is the equivalent of a population with uniform fitness, and modeled fitness gains relative to this population. We examined the clonal interplay of superdriver and driver mutations as they accumulate over time, by varying their selective advantages In addition, we propose a simple analytical approximation for the waiting time to a set of superdriver and driver mutations.

### Superdrivers dominate clonal evolution

**Fig. 2:**
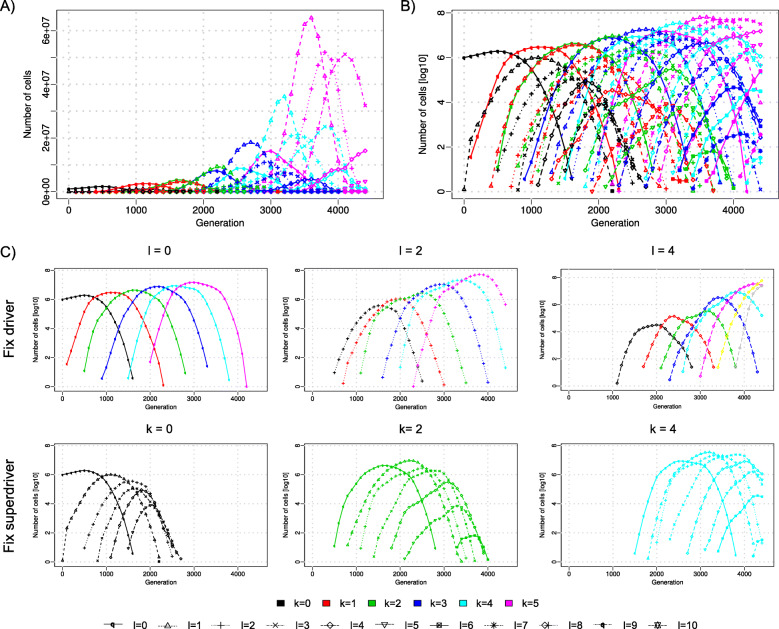
Traveling Gaussian waves of clonal expansions of superdrivers and drivers in an exponentially growing tumor cell population. **a** Number of cells with a given configuration of *k* ∊ {0, …, 5} superdriver and *ℓ* ∊ {0, …, 10} driver mutations shown by different colors and symbols, respectively. **b** Same as (**a**), but on a logarithmic scale. **c** Subsets of curves of panel (**b**) with fixed number of either drivers or superdrivers, revealing that tumorigenesis is dominated by superdriver accumulation. While clones with *ℓ* = 0 drivers have comparable frequency regardless of the number of superdrivers, the frequency of clones with *ℓ* = 4 drivers increases steeply with the number of superdrivers (upper panel “Fixed driver”). Additional drivers reach only low frequencies (lower panel “Fixed superdriver”) and eventually die out. All cell counts are averages over 50 replicates. For generating this figure, superdriver selection factor was set at *c* = 2 and driver selection at *s* = 0.01

The number of cancer cells with a given number of superdriver and driver mutations over time follows approximately a Gaussian distribution (Fig. 2a-b). We averaged the frequencies across all replicates and observed that superdrivers and drivers accumulate fundamentally differently. While the number of accumulated superdrivers in the population of cells globally increases constantly over time, the number of accumulated drivers varies in a periodic fashion conditioned on the number of superdrivers (Fig. 2c). Thus, tumor evolution is mainly driven by superdriver accumulation, which can be described as a traveling wave [21]. Within the superdriver waves, additional drivers accumulate in patterns of shorter traveling waves. Although additional driver mutations provide further increase in fitness, the clones harboring both superdrivers and drivers eventually become extinct, as new clones with more superdrivers arise. The outperformance of drivers is also reflected in that clones with higher numbers of drivers, irrespective of the number of superdrivers, only reach relatively low frequencies. Importantly, from the same reasons, harboring additional drivers does not seem to lead to higher frequencies of clones that have the same number of superdriver mutations.

### Shift of evolutionary dynamics

**Fig. 3:**
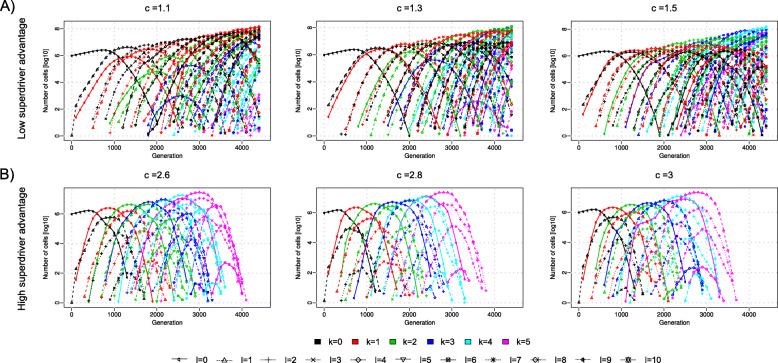
Change in evolutionary dynamics. **a** Low superdriver selection allows clones with the same number of superdrivers to exist for a long period of time. Every wave of additional superdriver mutations entails many driver waves with the same number of superdrivers, all of which reach high frequencies. **b** High superdriver selection changes the dynamics. Every superdriver wave now entails only a few additional drivers of low frequency and superdriver accumulation occurs quicker. All figures display the average frequency of 50 replicates. Superdriver selection was varied at *c* ∊ {1, 1.1, 1.3, 2.6, 2.8, 3}, while driver selection was set to *s* = 0.01. Only clones with *k* ∊ {0, …, 5} superdriver and *ℓ* ∊ {0, …, 10} driver mutations are displayed to facilitate comparison

The superdriver fitness increase parameter *c* controls the evolutionary dynamics of the growing tumor cell population. As *c* increases, the population transforms from a population that evolves mainly via clonal expansions of drivers (Fig. 3a) to a population that is driven by clonal expansions of superdrivers (Fig. 3b).

**Fig. 4:**
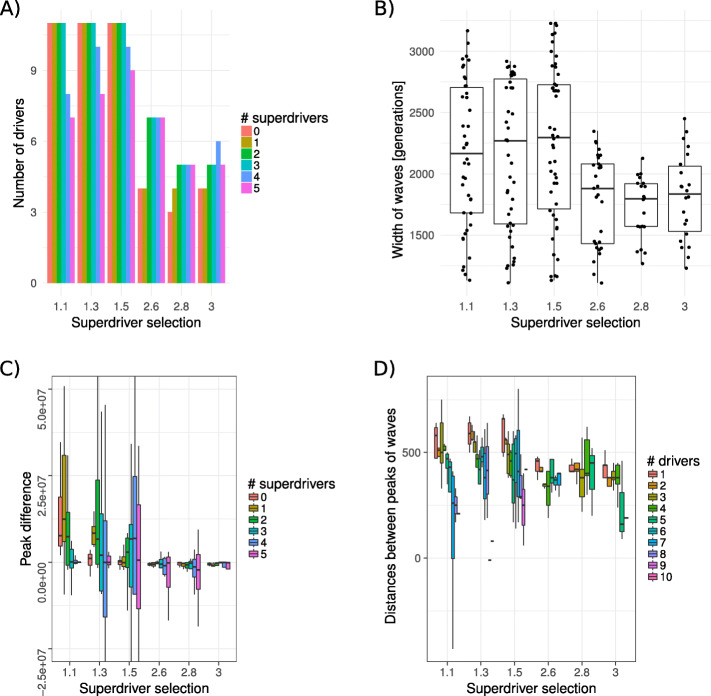
Analysis of traveling wave patterns. Based on average frequencies over 50 replicates, four statistics were measured for different fitness configurations: the number of driver waves within an superdriver evolution, width of waves, the difference in height between consecutive waves, and the number of generations between consecutive waves. We compared low superdriver selection (c ∊ {1.1, 1.3, 1.5}) and high superdriver selection (c ∊ {2.6, 2.8, 3.0}). Furthermore, we compared results between *k* ∊ {0, …, 5} superdriver and *ℓ* ∊ {0, …, 10} driver mutations. For all results, we fixed driver selection at *s* = 0.01. **a** Higher *c* leads to fewer driver waves conditioned on *k* and **b** all waves that span at least 500 generations tend to exist for fewer generations with less variance. **c** The difference in the maximum frequency of subsequent driver waves conditioned on *k* is lower for higher values of *c*; and **d** with higher *c* and conditioned on *l*, less generations lie between subsequent peaks of superdriver waves, suggesting that the maximum frequency of superdriver clones is reached in less generations

Stronger superdriver selection (2.6 < *c* < 3) entails fewer driver waves within a superdriver wave (Fig. 4a); each of those superdriver waves shows lower dispersion indicating that driver waves die out more quickly (Fig. 4b) and clones with a given number of superdrivers exist for a shorter period of time. Furthermore, peaks of subsequent driver waves (conditioned on the number of superdrivers) are closer in time for higher superdriver selection (Fig. 4c). Similarly, the maximum frequency of superdriver waves (conditioned on the number of drivers) tends to be reached in fewer generations when superdriver selection is higher (Fig. 4d). Consequently, higher superdriver factor *c* results in lower frequencies of new clones that do not carry superdriver mutations. In contrast, lower superdriver selection (1.1 < *c* < 1.5) leads to more driver waves within each superdriver wave, which also become wider. In this scenario, cells with a given number of superdrivers exist for more generations and hence superdriver accumulation occurs slower.

### Traveling wave analytics

To further investigate the above simulation results, which are based on averages across 50 replicates, we fixed driver selection at 0.01 and fitted quadratic polynomials for every wave in every individual replicate. From the fitted models, we extracted three parameters: location (i.e., generation), height (i.e., frequency), and curvature (i.e., dispersion). We observed that location followed a sigmoidal curve as a function of superdriver selection, increasing both with the number of superdriver and driver mutations (Supplementary Fig. 1). Fitted height showed a different pattern. While superdriver waves without driver mutations had higher frequency when superdriver selection was high, superdriver waves with one or two driver mutations had relatively lower frequency when superdriver selection was high (Supplementary Fig. 2). These clones were likely outcompeted by fitter clones with only superdriver mutations. For curvature, a similar pattern was observed. Without driver mutations, curvature of superdriver waves had consistently higher negative magnitude (i.e., narrower curve) for high superdriver selection compared to low superdriver selection (monotonic decrease), indicating higher growth rate of superdriver clones. Curvature of superdriver waves with two driver mutations and at least one superdriver mutation tended to be higher for high superdriver selection (non-monotonic increase, Supplementary Fig. 3) and hence the growth rate of superdriver clones was lower compared to the situation when the superdriver selection was high.

### Waiting time analysis

**Fig. 5:**
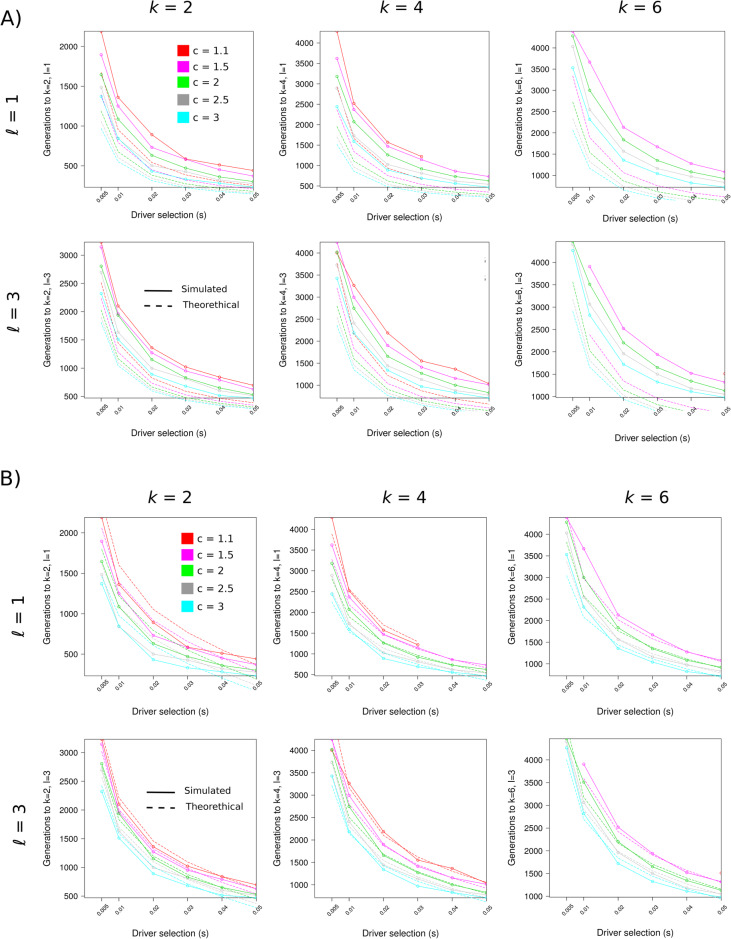
Comparison between simulated waiting times and the theoretical sum approximation. **a** We examined how the expected waiting time τ_*kℓ*_ to *k* superdrivers and *ℓ* drivers, as calculated from the sum of the individual waiting times (dashed), agree with the empirical waiting times from the simulations (solid). While for lower *k* and *ℓ* the agreement is generally high, the agreement decreases as *k* and *ℓ* increase. **b** Improved waiting time approximation, particularly for higher *k* and *ℓ*. We modeled the deviation between the simulated waiting times and the analytically approximated waiting times using a regression model with driver fitness, superdriver factor, and numbers of driver and superdriver mutations to wait for as covariates. The regression revealed that driver fitness parameter was the predictor with the largest effect size

Our simulations revealed that tumorigenesis in the presence of both superdrivers and drivers is driven by clonal expansions of superdrivers and hence may be approximated by traveling waves of clonal expansion [21]. Moreover, within the evolution of each superdriver wave, shorter traveling waves of drivers arise and provide additional selective advantages. These results motivate the hypothesis that the waiting time τ_*kℓ*_ to *k* superdriver and *ℓ* driver mutations can be decomposed into two independent components: *T*_*k*_^*S*^, the waiting time to *k* superdrivers, and *T*_*ℓ*_^*D*^, the waiting time to *ℓ* drivers. Our simulations indeed support that τ_*kℓ*_ ≈ *T*_*k*_^*S*^ + *T*_*ℓ*_^*D*^, i.e., the waiting time of a (*k*, *ℓ*) mutant is approximately the sum of the individual waiting times *T*_*k*_^*S*^ and *T*_*ℓ*_^*D*^ (Fig. 5a). We compared these predicted waiting times τ_*kℓ*_, computed as the sum of the individual waiting times, to the waiting times resulting from the simulation of the Wright-Fisher model, and found high concordance for low *k* and *ℓ*, and moderate concordance for large *k* and *ℓ* (Fig. 5a).

Furthermore, we observed that our theoretical approximation tends to slightly underestimates the simulated waiting times. To both understand the source of this deviation and correct for it, we empirically learned the residuals using a linear regression model, regressing them on the covariates *s*, *r*, *k*, and *ℓ* (i.e., driver fitness, superdriver fitness, number of driver mutations waited for, and number of superdrivers waited for, respectively). As shown in Fig. 5b, this extension improved the approximation (adjusted *R*^2^ = 0.77, F-statistic = 3739, *p*-value = 2.2 × 10^− 16^). From the regression analysis, we concluded that driver selection is the primary factor accountable for the deviation of the approximation to the simulation; the higher the selective advantage *s* of driver mutations, the larger is the gap between simulation and analytical approximation. All other remaining covariates contributed to the deviation significantly as well, but with smaller effect sizes (Supplementary Table 1). To further understand the relative effects of superdriver and driver fitness (i.e., *r* / s = c), we also tested a linear regression model that contained *s*, *c*, *k*, and *ℓ* as predictors (Supplementary Table 2). The driver fitness had the largest predictive value in this setting as well. The adjusted R^2^ of this model was similar to the first regression model (adjusted *R*^2^ = 0.78, F-statistic = 3814, *p*-value = 2.2 × 10^− 16^).

## Discussion

In this study, we have extended the simple Wright-Fisher model of tumor progression introduced in [21], which assumes that all driver mutations confer the same selective advantage, by introducing superdrivers, an aggressive type of driver mutation with higher selective advantage than ordinary drivers. This concept is consistent with earlier observations that the fitness landscape in many organisms may be characterized by a few very rare but highly advantageous mutations [27, 28]. However, quantitative models to study the interaction between these two classes of mutations, superdrivers and drivers, particularly in the context of cancer progression, have not been available to date. Thus, the present model is a first step towards modeling more complex fitness landscapes, where different mutations harbor different fitness effects. Using extensive simulations, we found that populations of tumor cells evolve in global clonal expansions of superdrivers and periodic expansions of drivers. This process can be described by a traveling Gaussian wave approach, which we utilized to approximate the expected joint waiting time to a certain number of superdrivers and drivers.

Our results suggest that tumorigenesis is dominated by superdriver mutations. We demonstrated that the number of driver waves within a superdriver wave is controlled by the superdriver selection parameter *c*. Increasing *c* will lead to fewer driver waves of low frequency and to quicker accumulation of superdrivers in the entire population of cells. Hence, the superdriver fitness increase parameter *c* triggers an evolutionary shift from a population that evolves through clonal expansions of drivers to a population that evolves through clonal expansions of superdrivers. This dominance of superdrivers could explain how the selective advantage becomes strong enough to noticeably change the evolutionary dynamics of driver mutations [29–31]. These results are further supported by our analysis of the distribution of parameters extracted after fitting quadratic polynomials to the traveling mutant waves (location, height, and curvature). In particular, the rate at which clones grow tends to be higher when superdriver selection is high, suggesting that superdriver fitness accelerates tumorigenesis. Furthermore, the absolute height of traveling waves is generally lower when superdriver selection is high, indicating that competitive clones with higher fitness more quickly outperform clones with lower fitness.

The fitness effects of mutations, quantified by the superdriver and driver fitness parameters *c* and *s,* also determine the generations required for the appearance of genotypes with certain numbers of mutations [32]. In general, cells with higher numbers of drivers only reach low frequencies, as they become extinct when cells with an additional superdriver arise. This periodic outperformance of drivers confirms that harboring one additional superdriver is selectively more advantageous than the accumulation of additional drivers, as was suggested especially for early-stage cancers [33]. This finding is in line with previous clinical observations that early mutations of highly selective genes, such as *APC* or *KRAS*, strongly favor the onset of cancer [26, 34, 35].

The simulated traveling Gaussian wave patterns suggested that the waiting times to superdrivers and drivers can be decoupled. We further showed that the waiting time to *k* superdrivers and *ℓ* drivers can be approximated by the sum of the individual waiting times, which renders high concordance to the empirical simulations for small *k* and *ℓ*. For increasing *k* and *ℓ*, the agreement to our simulations decreases, uniformly underestimating the simulated waiting times (especially for increasing driver selection *s*). As this discrepancy increases for larger number of mutations we conclude that the evolution of superdrivers and drivers can be decoupled primarily for early-stage tumors and needs to be adjusted for the variance observed in late-stage tumors.

To better understand the source of this discrepancy, we empirically learned the residuals between the theoretical approximation and the simulations by using linear regression. In addition to improving the waiting time approximation, the regression revealed that, from the included covariates, driver selection had the highest predictive power. One reason for this effect could be that clones with larger numbers of driver mutations evolve only very late, if at all, as it is more advantageous for a clone to acquire additional superdrivers. This means that the speed and possibly shape of driver waves are likely not constant, violating some of the assumptions of the approximation thereby leading to discrepancies between the estimated and simulated waiting times. In particular, the width of the driver wave is likely not constant; however, as we showed that driver mutations occur periodically within a superdriver wave, this parameter is negligible particularly for large values of *c*.

In an additional regression analysis, we found that the ratio between driver fitness and superdriver increase factor was a significant predictor for the approximation error as well. This model performed similarly to the model with driver fitness and superdriver factor included separately. The two regression models suggest that even though both effects are significant, the effect of driver fitness alone on the deviation between simulation and approximation is smaller than the effect of the relative difference between superdriver and driver fitness.

It is reasonable to assume that the number of expected superdrivers *k* is very low, considering that a small number of potent driver mutations have been described in the literature [2]. A recent study suggests that only three driver mutations may be sufficient to drive cancer in lung and colon [36]. Examples include *TP53* point mutations or *EGFR* copy number variations across multiple cancer types [37, 38], or *POT1* depletion, a particularly aggressive alteration that is suggested to dramatically accelerate tumorigenesis in T cell lymphoma [39]. In addition, empirical observations suggest that the selection intensity of such mutations is significantly higher than the vast majority of alternative mutations found from sequencing data [28], reinforcing the need to account for the much higher relative importance of those mutations in models of tumorigenesis. Alternatively, the number of drivers *ℓ* can be expected to be higher than the number of superdrivers *k*, even though this parameter is also estimated to be below ten, depending on the type of tissue [40]. In addition, some related studies suggest that tumorigenesis may be driven by mutations with relatively low fitness increase. For example, a review by Castro-Giner et al. discusses the concept of ‘mini-drivers’ [41], i.e., selectively advantages mutations with relatively weak fitness increase. Their drivers and mini-drivers are covered by our model as superdrivers and drivers, respectively. Mini-drivers are claimed to be able to drive tumorigenesis even in the absence of drivers, a situation that would arise in our simulations only in the absence of superdrivers (*k* = 0) or by assuming a lower superdriver mutation rate. Future studies could investigate whether the mini-driver concept is reflected by our model when superdrivers are allowed to occur only very rarely.

One limitation of our study is that every simulation has to be terminated after a certain amount of time has passed, as all simulations of tumorigenesis have to select a viable time range to allow for sufficient progression time [42–44]. In our study, we chose to terminate simulations at 4500 generations, as this number corresponds approximately to a tumor development of 12 years, which is a sufficiently large time period that has been used previously [21]. For example, our results in Fig. 4a suggest that with low superdriver selection (i.e., 1.1 ≤ *c* ≤ 1.5), the number of drivers decreases as the number of superdrivers decreases; however, this could potentially happen because drivers that occur in very late generations (i.e., in generation 4000 and higher) cannot reach high enough frequencies before the simulations are terminated.

Additional limitations of the model include the basic assumption that every mutation provides a constant fitness gain, depending only on whether a superdriver or driver loci was hit. Clearly, for biological systems however, the fitness gain of a mutated gene may vary even for the same mutation in different individuals and across cancer types [27], and hence the superdriver and driver fitness parameters *c* and *s* should be regarded as averages of fitness increases. Our model could, however, be extended by sampling c and s from a probability distribution. Moreover, we modeled all loci to be independent of each other, an approximation of the true (but unknown) underlying fitness landscape which can have interactions, known as epistasis [45, 46]. Also, our model ignored the we neglected spatial heterogeneity of solid tumors, which can significantly impact clonal dynamics slow down tumor progression by clonal interference [47]. Finally, our model is time-discrete in how clonal evolution occurs. Even though discrete models have served extensively to reveal evolutionary patterns in previous studies [14, 21, 22, 48–50], they represent an abstraction of continuous-time biological systems. Future studies could extend our results by employing continuous model choices.

The justification for simulating tumor evolution based on superdrivers and drivers type of alterations alone, is that understanding simple mutational processes is the foundation for understanding more complex models of tumorigenesis. Future studies should investigate the dynamics of drivers and superdrivers in the presence of other mutation types, or under different parameter landscapes and could be compared to in vivo data [51–53]. Similar to our study nevertheless, Datta et al. [22] extended the model in [21] and showed that deleterious mutations have little effect on tumorigenesis unless driver selection is very weak. Since superdrivers are mutations with high fitness advantage, it is very likely that, under reasonable assumptions, deleterious mutations will be extinguished from the population during tumor progression and not accumulate in later stages of tumorigenesis [40]. In addition, the simulations in Datta et al. [22] included a mutator phenotype [54] with elevated mutation rate. Their analyses suggested that the mutator phenotype could evolve only in situations with low driver selective advantage. Future work could therefore determine whether, and if so, in which case, superdrivers suppress the development of a mutator phenotype. In contrast, a reduced mutation generally leads to genomic stability and hence, it can be expected that superdrivers will become rare and gradually lose their dominance in favor of driver dominance.

Undoubtedly, patient tumors are much more complex than represented through the mathematical model employed here, and exhibit various additional biological properties, such as, among many others, the presence of immune surveillance, epistasis, cellular competition for resources, cell-cell signaling, environmental factors. Nevertheless, mathematical models describing an evolutionary environment with selection and fitness allow, through their simplicity, to investigate focused research question towards specific parameters of interest. This led, for example, Bozic et al. 2010 to narrow down possible selection rates [14], and other studies to identify candidate driver genes for drug discovery [35], as well as investigate the clinical [55–57] and biological impact [58–60] of driver genes. Our model contributes to addressing such biomedical questions by allowing other researchers to better understand the dynamics of genetically-driven tumorigenesis.

## Conclusion

In summary, our work presents a mathematical model to study the interplay of superdriver and driver mutations in tumorigenesis. By simulating under this model, we demonstrated that superdriver mutations, although more unlikely to occur than driver mutations, are the dominant evolutionary force driving the progression to cancer. Moreover, we found that, for small numbers of mutations, the waiting time to a set of superdrivers and drivers can be approximated by the sum of the individual waiting times.

## Supplementary information

**Additional file 1: Supplementary Figure 1.** Location (i.e., generation) extracted from fitting quadratic polynomials for all 50 replicates separately at driver selection *s* = 0.01. The figure displays the distribution of location for waves with 0–3 superdriver mutations (columns, S0-S3) and 1–2 driver mutations (rows, D1 and D2). Generally, locations follows a sigmoid curve and is shorter for high superdriver selection *c*.

**Additional file 2: Supplementary Figure 2.** Height (i.e., frequency) extracted from fitting quadratic polynomials for all 50 replicates separately at driver selection *s* = 0.01. Columns and rows are number of superdriver and driver mutations, respectively: 0–3 superdriver mutations (S0-S3) and 0–2 driver mutations (D0 -D2). For waves with two driver mutations, height of waves is significantly lower when superdriver selection is high.

**Additional file 3: Supplementary Figure 3.** Curvature (i.e., dispersion) extracted from fitting quadratic polynomials for all 50 replicates separately at driver selection *s* = 0.01. Columns and rows are number of superdriver and driver mutations, respectively: 0–3 superdriver mutations (S0-S3) and 0–2 driver mutations (D0 -D2). Only for waves with no driver mutations, curvature of all superdriver waves are lower when superdriver selection is high. For waves with two driver mutations, curvature is slightly higher when superdriver selection is high.

**Additional file 4: Supplementary Table 1.** First linear regression model used to predict the deviation between the simulated waiting times and the analytical approximation.

**Additional file 5: Supplementary Table 2.** Second linear regression model used to predict the deviation of the simulated waiting times and analytical approximation.

## Data Availability

Code to generate the simulations of this publication will be available on https://github.com/pgrossmann/Superdriver_Clonex. Code to reproduce the analysis of this publication will be available at https://github.com/pgrossmann/Superdriver_Analysis.

## Abbreviations

DNA: Deoxyribonucleic acid
*TP53*: Gene coding for tumor protein p53
*EGFR*: Gene coding for epidermal growth factor receptor
*KRAS*: Gene coding for the K-Ras signaling protein of the RAS/MAPK pathway
*POT1*: Gene coding for protection of telomeres 1
*APC*: Gene coding for adenomatous polyposis coline
