# Supplementary figures and images for: Clonal evolution driven by superdriver mutations
Source: BMC Evol Biol. 2020 Jul 20;20:89. doi: 10.1186/s12862-020-01647-y (PMC7370525; doi:10.1186/s12862-020-01647-y)

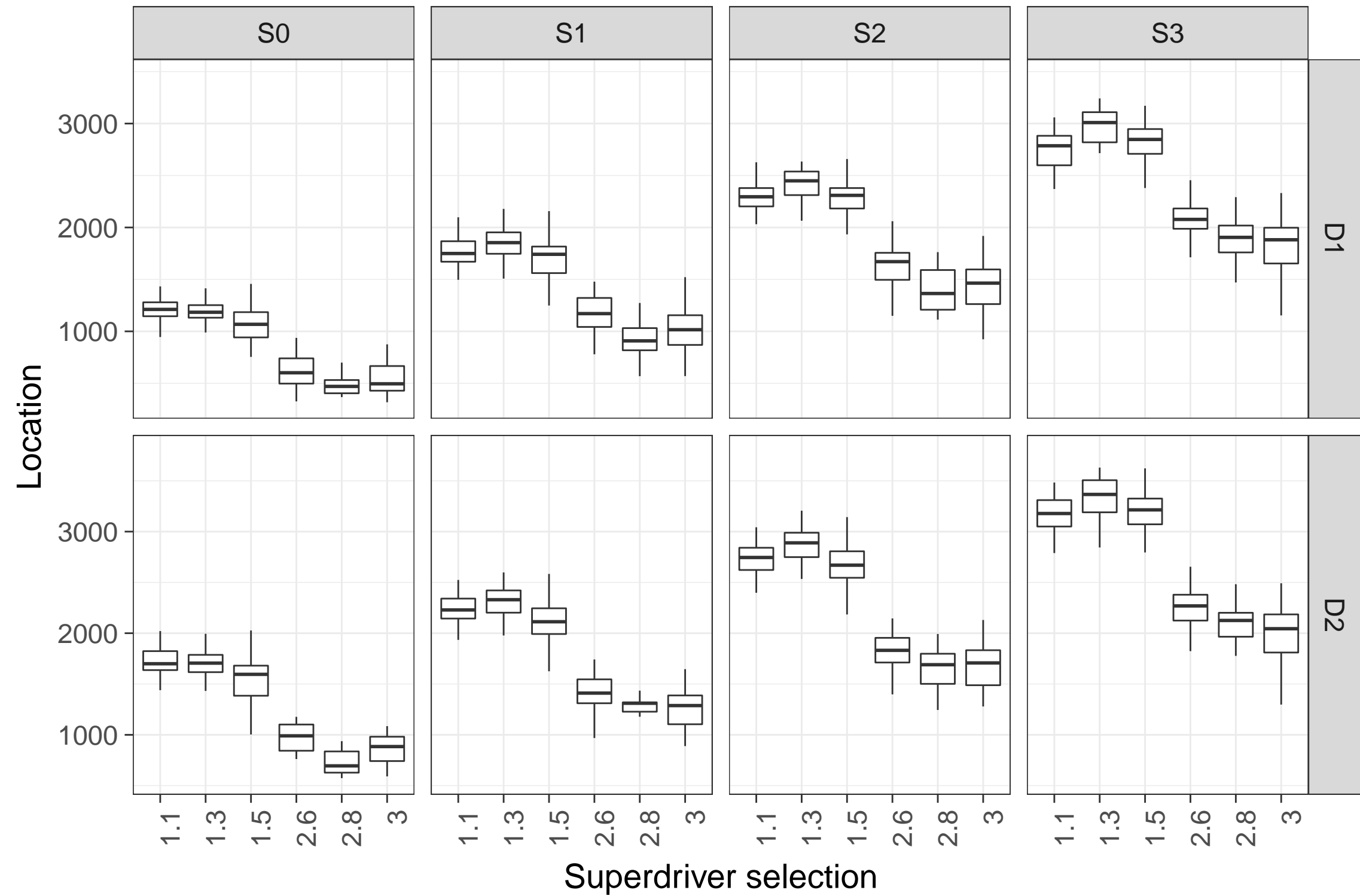

Supplement: Supplementary file 1 — Additional file 1: Supplementary Figure 1. Location (i.e., generation) extracted from fitting quadratic polynomials for all 50 replicates separately at driver selection s = 0.01. The figure displays the distribution of location for waves with 0–3 superdriver mutations (columns, S0-S3) and 1–2 driver mutations (rows, D1 and D2). Generally, locations follows a sigmoid curve and is shorter for high superdriver selection c. [file 12862_2020_1647_MOESM1_ESM.pdf]

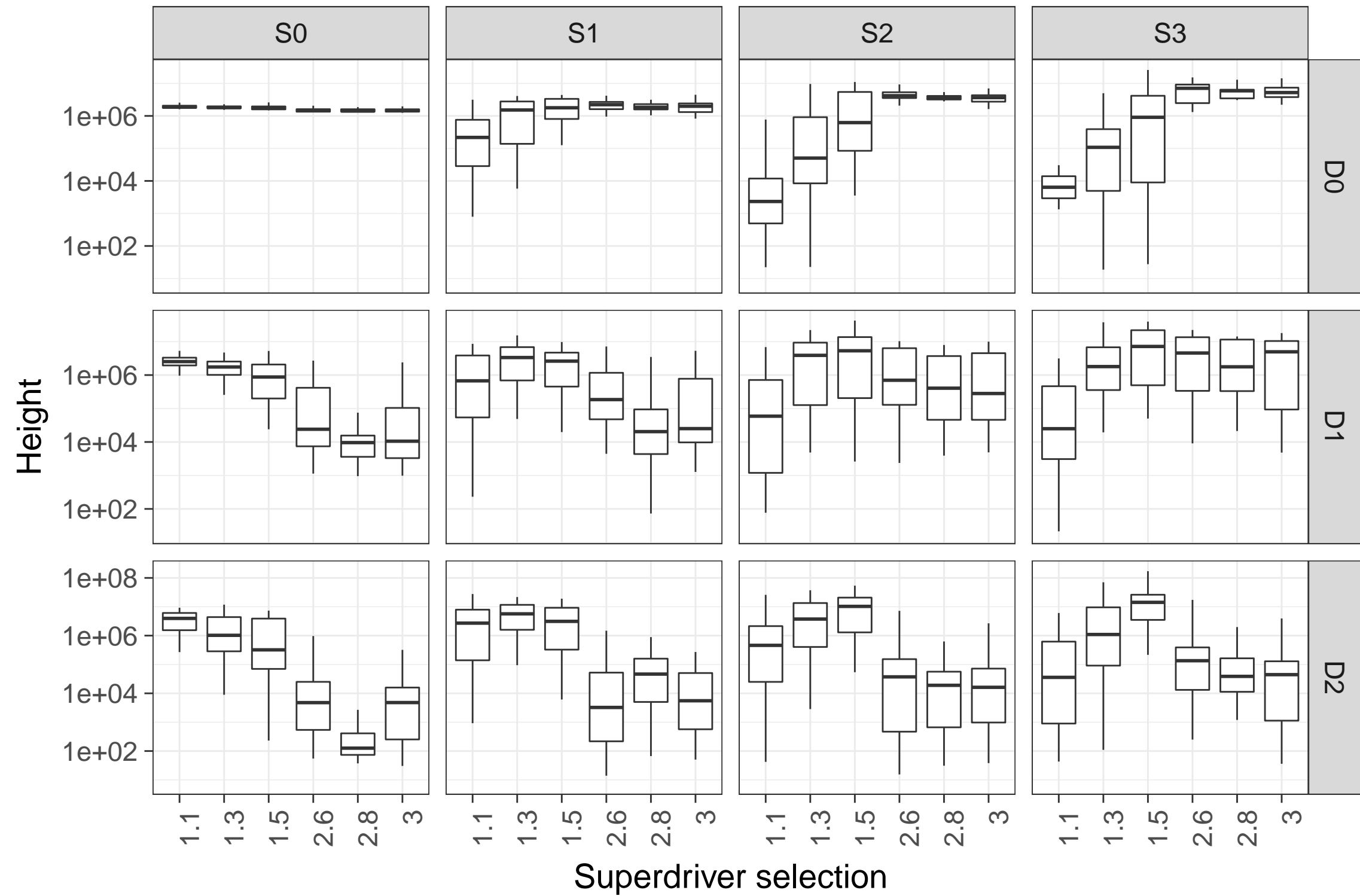

Supplement: Supplementary file 2 — Additional file 2: Supplementary Figure 2. Height (i.e., frequency) extracted from fitting quadratic polynomials for all 50 replicates separately at driver selection s = 0.01. Columns and rows are number of superdriver and driver mutations, respectively: 0–3 superdriver mutations (S0-S3) and 0–2 driver mutations (D0 -D2). For waves with two driver mutations, height of waves is significantly lower when superdriver selection is high. [file 12862_2020_1647_MOESM2_ESM.pdf]

Curvature

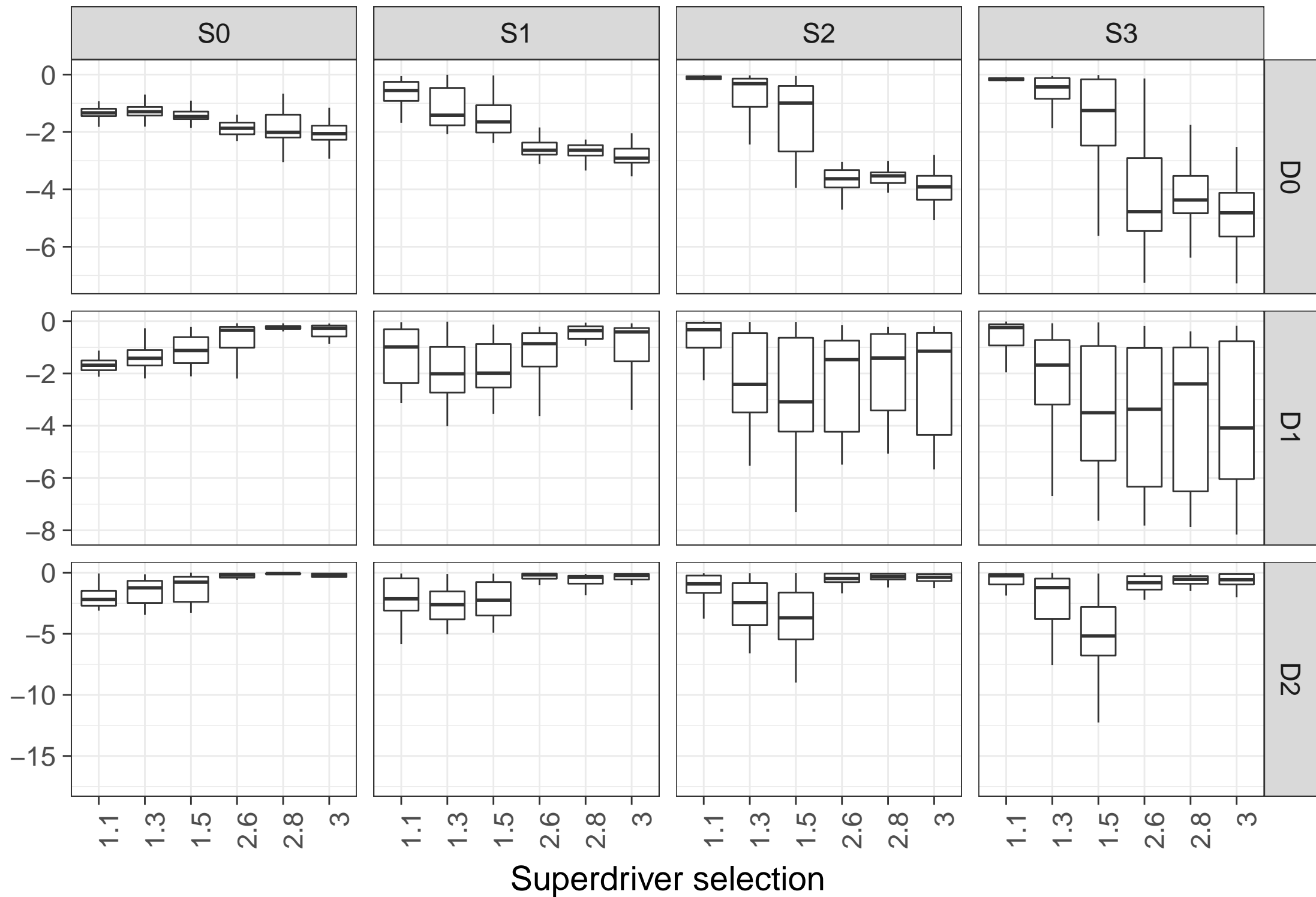

Supplement: Supplementary file 3 — Additional file 3: Supplementary Figure 3. Curvature (i.e., dispersion) extracted from fitting quadratic polynomials for all 50 replicates separately at driver selection s = 0.01. Columns and rows are number of superdriver and driver mutations, respectively: 0–3 superdriver mutations (S0-S3) and 0–2 driver mutations (D0 -D2). Only for waves with no driver mutations, curvature of all superdriver waves are lower when superdriver selection is high. For waves with two driver mutations, curvature is slightly higher when superdriver selection is high. [file 12862_2020_1647_MOESM3_ESM.pdf]
