# Supplementary material for: Clonal evolution driven by superdriver mutations
Source: BMC Evol Biol. 2020 Jul 20;20:89. doi: 10.1186/s12862-020-01647-y (PMC7370525; doi:10.1186/s12862-020-01647-y)
Supplement: Supplementary file 4 — Additional file 4: Supplementary Table 1. First linear regression model used to predict the deviation between the simulated waiting times and the analytical approximation. [file 12862_2020_1647_MOESM4_ESM.docx]

**Supplementary Table 1:** First linear regression model used to predict the deviation between the simulated waiting times and the analytical approximation.

| **Covariate** | **Estimate** | **Std. Error** | ***t* value** | **p-value** |
| --- | --- | --- | --- | --- |
| Intercept | 402.49 | 8.21 | 49.02 | < 1.0e-308 |
| *s*  (driver fitness) | -7012.81 | 332.01 | -21.12 | 2.3e-94 |
| *r*  (superdriver fitness) | -3323.33 | 159.87 | -20.79 | 1.3e-91 |
| *k*  (number of superdriver mutations to wait for) | 117.05 | 1.56 | 54.93 | < 1.0e-308 |
| *ℓ*  (number of driver mutations to wait for) | 58.52 | 1.07 | 54.93 | < 1.0e-308 |
