# Supplementary material for: Clonal evolution driven by superdriver mutations
Source: BMC Evol Biol. 2020 Jul 20;20:89. doi: 10.1186/s12862-020-01647-y (PMC7370525; doi:10.1186/s12862-020-01647-y)
Supplement: Supplementary file 5 — Additional file 5: Supplementary Table 2. Second linear regression model used to predict the deviation of the simulated waiting times and analytical approximation. [file 12862_2020_1647_MOESM5_ESM.docx]

**Supplementary Table 2:** Second linear regression model used to predict the deviation of the simulated waiting times and analytical approximation.

| **Covariate** | **Estimate** | **Std. Error** | ***t* value** | **p-value** |
| --- | --- | --- | --- | --- |
| Intercept | 609.97 | 12.99 | 46.96 | < 1.0e-308 |
| *s*  (driver fitness) | -13178.56 | 165.92 | -79.43 | < 1.0e-308 |
| *c*  (superdriver fitness factor) | -111.19 | 4.94 | -22.52 | 2.5e-106 |
| *k*  (number of superdriver mutations to wait for) | 118.41 | 1.55 | 76.38 | < 1.0e-308 |
| *ℓ*  (number of driver mutations to wait for) | 57.52 | 1.06 | 54.07 | < 1.0e-308 |
